# Osmotic Stress Blocks Mobility and Dynamic Regulation of Centriolar Satellites

**DOI:** 10.3390/cells7070065

**Published:** 2018-06-22

**Authors:** Julie C. Nielsen, Cathrine Nordgaard, Maxim A. X. Tollenaere, Simon Bekker-Jensen

**Affiliations:** Center for Healthy Aging, Department of Cellular and Molecular Medicine, University of Copenhagen, Blegdamsvej 3B, DK-2200 Copenhagen N, Denmark; juliecn@sund.ku.dk (J.C.N.); nordgaard@sund.ku.dk (C.N.)

**Keywords:** centriolar satellites, cell stress, osmotic stress, MAP kinase signalling, p38, MK2

## Abstract

Centriolar satellites (CS) are small proteinaceous granules that cluster around the centrosome and serve as cargo vehicles for centrosomal proteins. It is generally accepted that CS support a number of canonical and specialized centrosome functions. Consequently, these highly dynamic structures are the target of regulation by several cellular signalling pathways. Two decades of research have led to the identification of a large number of molecular components and new biological roles of CS. Here, we summarize the latest advances in the continuous efforts to uncover the compositional, functional, dynamic and regulatory aspects of CS. We also report on our discovery that osmotic stress conditions render CS immobile and insensitive to remodelling. Upon a range of p38-activating stimuli, MK2 phosphorylates the CS component CEP131, resulting in 14-3-3 binding and a block to CS formation. This normally manifests as a rapid cellular depletion of satellites. In the case of osmotic stress, a potent inducer of p38 activity, CS translocation and dissolution is blocked, with the net result that satellites persist in an immobile state directly adjacent to the centrosome. Our results highlight a unique scenario where p38 activation and CS depletion is uncoupled, with potential implications for physiological and pathological osmotic stress responses.

## 1. Introduction

**Figure 1:**
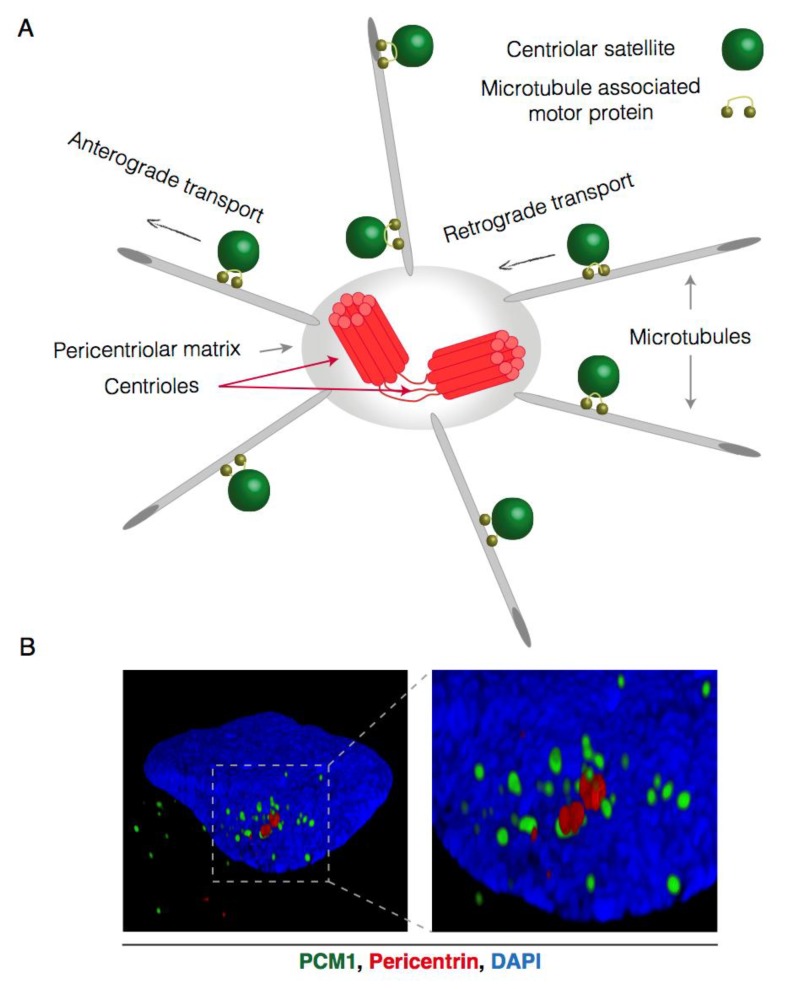
Microtubule-dependent and centrosome-oriented transport of centriolar satellites. (**A**) Schematic representation of centriolar satellite (CS) transport. CS gravitate around the centrosome and are connected to the microtubule network through microtubule-associated motor proteins. These motor proteins facilitate the stepwise movement of centriolar satellites towards (retrograde) or away from (anterograde) the centrosome; (**B**) Reconstructed 3D-Structured Illumination Microscopy (3D-SIM) images of centriolar satellites in interphase U2OS cells. Centriolar satellites are visualized by PCM1 staining (green), the centrosome is visualized by pericentrin (red) staining and the nucleus (DAPI) in blue.

Centriolar satellites (CS) are small, microscopically visible, proteinaceous granules that gravitate around the centrosome (Figure 1A,B) [1,2,3,4]. After more than two decades of research it has become clear that CS are functionally and spatially linked to the centrosome and contain many proteins which are also found to localize to the centriolar and pericentriolar regions as well as the ciliary transition zone [5,6,7,8]. In interphase cells, CS are linked to the microtubule network by motor proteins, which promote their movement towards or away from the centrosome [2,9,10,11]. Thus, in cells where the microtubule network is disturbed, or depolymerized by drugs such as nocodazole, CS are randomly scattered throughout the cytosol [4]. CS are both exquisitely dynamic and sensitive to various intra- and extracellular cues, such as the cell-cycle and exposure to stressors, that can directly impact on the molecular composition, localization and function of these structures. As CS contain numerous structural and regulatory centrosomal components, they are ideally positioned to communicate perturbations or cell-cycle status to the centrosome. Hence, their molecular function with regard to canonical and specialized centrosome functions has formed the basis of numerous studies. It is generally recognized that CS support a variety of centrosomal processes, including centrosome biogenesis, cytokinesis and ciliogenesis (reviewed in [5,7]). To this end, they either serve as transport vehicles that deliver protein cargo to the centrosome and ciliary transition zone, or as sequestration platforms that buffer the access of proteins to these cellular compartments [12]. Dysfunctional centrosomes and/or cilia can have detrimental effects on human health and are implicated in various disorders including primordial dwarfism, primary microcephaly and a group of heterogeneous diseases collectively termed ciliopathies (e.g., Joubert syndrome, Oral–Facial–Digital syndrome and Bardet–Biedl syndrome) [13]. A large number of the mutations known to be causative for the aforementioned disorders localize to genes that code for proteins residing in CS, highlighting the critical role that these structures play in supporting centrosomal functions [5,11,13,14,15,16,17].

CS contain both positive and negative regulators of centrosome biogenesis and ciliogenesis. For example, OFD1, MIB1 and BCAP/ODF2L are factors that restrict cilium growth or ciliogenesis altogether [18,19,20]. Ciliogenesis has been suggested to require autophagic turnover of the CS-associated pool of OFD1, and inactivation of the E3 ubiquitin ligase MIB1. On the other hand, CS resident proteins such as TEX9 and CCDC112 are positive regulators of ciliogenesis and CS are required for the delivery of factors such as the BBSome component BBS4 and the small GTPase RAB8a to the ciliary transition zone [8,11,15,16,21,22,23]. Collectively, these events facilitate cilium biogenesis, ciliary vesicle trafficking and intraflagellar transport, respectively. CS have also been suggested to balance positive and negative regulators of centrosome duplication. In coordination with DNA replication, the centrosome is duplicated once per cell cycle [24]. Excessive or incomplete duplication or maturation of centrosomes may result in multipolar or fragmented spindles during mitosis, which can lead to aneuploidy and genome instability, a frequent hallmark of cancer cells [25,26]. Centrosome duplication factors such as CDK5RAP2, CEP152, WDR62, CEP63 and CDK2 are dependent on CS for their controlled access to the centrosome [17,27]. Hence, cells depleted of proteins that are critical for CS integrity or cargo recruitment, such as CEP131, KIAA0753/MNR, CCDC13 and PIBF1/CEP90, fail to assert stringent control over centrosome duplication. In contrast, other proteins, including the CS factor CCDC14, restrict centriole duplication, possibly by limiting CS mediated recruitment of CDK2 to the centrosome [17,28]. Given that CS have the capacity to transport, store, stabilize or locally destabilize a subset of centrosomal proteins, CS are ideally placed to orchestrate physical and functional remodelling of centrosomes. In line with such notion, research over the last 5 years has highlighted that the molecular composition, localization and structural integrity of CS is highly dynamic and amenable to dynamic regulation upon cell cycle transitions, acute stress conditions and inflammatory stimuli [6].

It is increasingly recognized that CS display considerable compositional heterogeneity. At present, more than 50 different proteins have been confirmed to reside in CS, either by co-localisation or direct interactions with CS scaffolding proteins, and some studies suggest that this number may increase to 200 [8,28]. Many of these putative CS components have been identified by mass-spectrometry based interaction and proximity screens, and in a proportion of cases these factors were shown to impact on centrosome biogenesis, ciliogenesis or the structural integrity and localization of CS [8,28]. Among the expanding inventory of CS components are numerous post-translational modifiers such as kinases, ubiquitin ligases and acetyltransferases [27,29,30,31,32]. Some of these may represent novel centrosomal cargo proteins, but may also serve as direct regulators of CS behaviour. We thus hypothesize that post-translational modification by these enzymes could hold the key to further understanding of cell cycle- and cell status specific functions of CS, as well as their heterogeneity, localization and fate.

### 1.1. Compositional Heterogeneity of Centriolar Satellites

It is becoming increasingly apparent that CS display considerable compositional heterogeneity and that CS can serve different functions depending on cell cycle status, activity of signalling pathways etc. [6]. Notably, CS composition and dynamics are exquisitely sensitive to a multitude of cellular stresses such as DNA damage [33], ribosome inhibition [30] and proteotoxic stress [19]. Numerous factors that have been shown to localize to CS, do not fully co-localize with other CS factors, and furthermore, not all CS-like structures containing centrosomal proteins contain the archetypical CS markers such as PCM1 and CEP131. UV-irradiation induced dissolution of CS, for example, does not affect the granular CS-like localization of OFD1 [19]. Similarly, BBS4 positive granules that do not contain PCM1 or CEP131 can be observed near the ciliary transition zone [15]. Such observations might reflect the dynamic exchange between the ciliary transition zone and CS, or indicate that not all CS contain the classical markers such as PCM1. The notion that CS are under strict control of the cell cycle was revealed in early studies of the behaviour and functions of these structures [1,2,4]. CS become virtually undetectable during cytokinesis, after which they re-assemble in early G1, likely through PLK4 mediated phosphorylation of PCM1 at Ser-372 [29]. More recent work has demonstrated that cell cycle state also impacts on the protein composition of CS. For example, USP9X is a de-ubiquitinase that controls PCM1 and CEP131 abundance, and which is incorporated into CS through direct interactions with CEP131 during S and G2 phases of the cell cycle [27]. More evidence in support of the hypothesis that not all CS share the same fate and function comes from the observations that a small population of PCM1- and SSX2IP-positive CS co-localize with GABARAP, a protein belonging to the ATG8 family of autophagy promoting and ubiquitin-like modifiers [34]. Indeed, a supporting role for CS in the formation of autophagosomes, through stabilisation and recruitment of GABARAP and the autophagic cargo receptor p62, has recently been suggested [34,35]. In this context, it is tempting to speculate that CS are able to sequester compromised or superfluous centrosomal proteins that are subsequently targeted for autophagic destruction. However, additional work suggested that PCM1 retains GABARAP in an inactive state at CS, and it is also plausible that CS act to adjust autophagic flux by releasing the “dormant” CS-associated GABARAP pool to the centrosome [12,36].

Adding to the complexity, CS also display asymmetrical spatial distribution around individual centrosomes as well as mother and daughter centrioles [37]. Monitoring the distribution of CS around split centrosomes in late G2 phase of the cell cycle highlights how in a sub-set of cells, CS are unequally distributed between the two centrosomes. Such observations hint that the “inheritance” of centriolar satellites can contribute to the asymmetric nature of certain cell divisions. Tozer and colleagues have recently shown that unequal association of CS with the mother and daughter centriole, respectively, contributes to asymmetrical cell division in neural progenitors [37]. This mechanism impinges on the sequestration of the ubiquitin E3 ligase MIB1 to CS surrounding the daughter centriole. In daughter cells that inherited the mother centriole, and where MIB1 could not be sequestered by CS, a secondary pool of MIB1 is released from the endoplasmic reticulum, augmenting Notch signalling at the plasma membrane and maintaining cells in their undifferentiated state [37,38]. While the molecular determinants underlying CS asymmetry between mother and daughter centrioles remain unknown, a role of CS in promoting interactions between HTT/huntingtin, HAP1, DISC1 and the calcium/calmodulin dependent kinase CaMKIIβ during neurogenesis have been reported [21,39,40,41]. These observations illustrate the diverse roles of CS in centrosome maintenance, ciliogenesis and beyond.

### 1.2. Centriolar Satellites and Autophagy

Cross-talk between the autophagy system and CS has been reported in a number of publications [18,34,35,42]. Autophagy plays a key role in maintaining cellular proteostasis by clearing non-functional, misfolded or otherwise undesirable proteins from the cell, and enabling replenishment of cellular energy pools upon starvation. Proteins and even entire organelles can be marked for autophagic destruction, which often occurs through posttranslational modifications (PTMs—e.g., ubiquitylation) and ensuing interaction with proteins from the ATG8 family through so-called LC3-interacting motifs (LIR) [43]. Already in 2010, PCM1 was proposed to interact with the ATG8-family proteins GABARAP and GABARAP2L [35]. This interaction was later consolidated by Joachim and colleagues, who identified a functional LIR in the carboxy-terminus of PCM1 and demonstrated that this sequence is responsible for targeting GABARAP to CS [34]. Interestingly, GABARAP association does not lead to autophagy-mediated turnover of the CS superstructure, but rather protects GABARAP from degradation by the proteasome. A similar role for CS in protecting TALPID3 from degradation has been proposed, and in this context, it is possible that CS function as protective storage containers to limit the turnover of specific proteins [12,44]. While GABARAP does not mark CS for degradation per se, other CS-components such as OFD1 can be removed from the CS structure and be degraded by the autophagy system [18]. In cycling cells, OFD1 is distributed between the centrosome and CS. The centrosomal pool of the protein is a prerequisite for proper centrosome biogenesis and ciliogenesis, and mutations in the OFD1 gene can lead to various ciliopathies including Oral-Facial-Digital syndrome, Joubert syndrome and nephronophthisis-related ciliopathy [14,45,46,47]. Conversely, the CS-associated pool of OFD1 has to be degraded to licence ciliogenesis. Through the association of PCM1 and authophagic regulators such as GABARAP, it is conceivable that CS are directly involved in delivering OFD1 to autophagosomes. How CS themselves are protected from being engulfed by authophagosomes and how OFD1 is specifically removed from the CS superstructure remains to be investigated.

### 1.3. Centriolar Satellites and the Ubiquitin System

**Figure 2:**
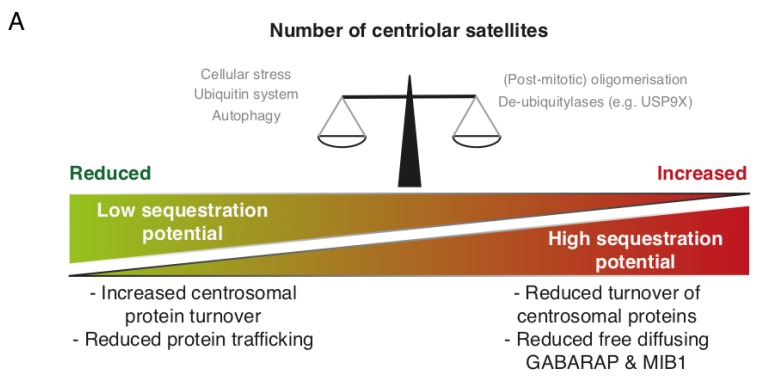

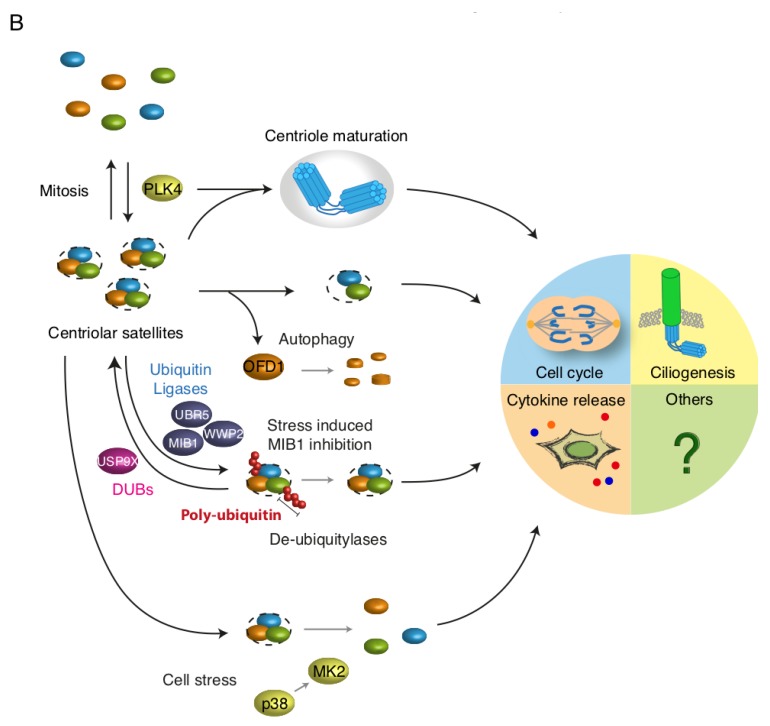
Dynamic regulation of centriolar satellites. (**A**) Regulation of centriolar satellites (CS) involves phosphorylation, ubiquitination and autophagy. CS may on the one hand act as sequestration hubs for proteins such as MIB1 and GABARAP, limiting their presence and activity elsewhere in the cell (e.g., the centrosome). On the other hand, disruption of CS impairs protein trafficking to the centrosome or basal body; (**B**) CS are susceptible to regulation by intra- and extracellular signals. CS are resolved during mitosis and are re-formed in G1 phase, possibly through PLK4 mediated phosphorylation. Autophagic destruction of CS-bound OFD1 precedes ciliogenesis. Several ubiquitin E3 ligases (WWP2, UBR5 and MIB1) ubiquitinate satellite components. De-ubiquitinases such as USP9X may counteract such modifications. Cell stress is accompanied by the deactivation of MIB1 which may stimulate ciliogenesis. Finally, cellular stress signalling interferes with *de novo* satellite formation through a mechanism dependent on the kinases p38 and MK2. The abundance, molecular composition and post-translational modifications of CS impact directly on centrosome-associated processes including centrosome maturation, ciliogenesis, mitosis and possibly cytokine secretion.

Protein ubiquitination is a common PTM that has diverse functions depending on the location, number and linkage type of ubiquitin conjugates. Ubiquitination occurs through a three-tiered hierarchical mechanism involving a ubiquitin activating enzyme (E1), a ubiquitin conjugating enzyme (E2) and a ubiquitin ligase (E3), with the latter component conferring substrate specificity. Along with detailed cell-biological experiments, large-scale proteomic investigations have revealed a number of E2, E3 and de-ubiquitinase (DUB) enzymes that interact with one or more core CS protein [8,19,27,48,49]. The E3 ligase MIB1 localizes to CS and can build K11-, K48- and K63-linked ubiquitin chains on target proteins [19,48,50]. MIB1 appears to have a wide repertoire of centrosomal targets and negatively impacts on centrosome duplication and ciliogenesis by promoting the proteasomal destruction of several targets. Among these are PLK4, a master regulator of centriole biogenesis, TALPID3, a protein functioning at the ciliary transition zone during ciliogenesis, and GABARAP, involved in formation of autophagosomes as mentioned above [34,44,50]. MIB1 was suggested to be sequestered by CS to prevent uncontrolled ubiquitination and degradation of centrosomal factors [12]. Hence, dysfunctional CS, or their complete absence, leads to increased proteasomal turnover of TALPID3 and GABARAP. A similar mechanism, where CS function to sequester MIB1, has been proposed to limit MIB1-driven Notch signalling after asymmetric division of neuronal progenitor cells [37]. The centrosomal substrates of MIB1 do not exclusively localize to the centrosome, but are also present at CS [5]. The substrate pools in this locale may be protected from degradation on account of the activity of resident DUBs such as USP9X and potentially others [27,49]. Interestingly, MIB1 also ubiquitinates core CS proteins, such as CEP131, PCM1 and CEP290 [19]. As the very same factors are required for recruitment of MIB1 to CS, such mechanisms could form the basis of a feedback loop that buffers the activity of MIB1. Overexpression of USP9X leads to increased CEP131 and PCM1 protein levels, excessive centrosome duplication and multipolar spindles, likely caused by an increased flux of centrosome duplication factors such as CDK2 [27]. *Vice versa*, depletion of USP9X also decreased cellular pools of CEP131 and PCM1. This was accompanied by reduced CDK2 accumulation at the centrosome and diminished centrosome over-duplication in S-phase arrested cells [27]. While it is unclear whether MIB1-mediated ubiquitination promotes the proteasomal turnover of CEP131, PCM1 and CEP290, the sequestration potential of CS may be balanced by the overall abundance of CS-proteins, their ubiquitination and USP9X-mediated deubiquitination (Figure 2A). MIB1 is inactivated following UV-irradiation induced cell stress through unknown mechanisms, an event that coincides with CS collapse (see below). MIB1 normally suppresses cilium formation, and it is possible that MIB1 inactivation and CS collapse collaborate to protect pro-ciliogenic factors from degradation.

### 1.4. Centriolar Satellites and Kinases

CS constitute a transport intermediate for centrosomal kinases such as NEK2a [51], CAMKIIβ [41] and CDK2 [17]. Thus, depletion of CS by knock-down of PCM1 leads to defective targeting of these kinases to the centrosome and compromises centrosome duplication. In addition, a number of kinases modify CS proteins and impact on the structural integrity, localization and function of CS (e.g., CDK1 [52], PLK1 [31], PLK4 [29] and MAPKAPK2/MK2 [30]). PLK4 is a key regulator of centriole duplication, the deregulation of which is associated with cancer-associated centrosome amplification [50,53,54]. Recent work suggests that PLK4 exerts similar control over CS abundance and localization [29]. Inhibition of PLK4 activity is accompanied by a reduction in CS numbers and arrested centriole duplication. Biochemical and mass-spectrometry based experiments revealed that PLK4 phosphorylates PCM1 in a region that mediates PCM1 oligomerisation and interactions with other CS-components. Introduction of a phosphorylation deficient PCM1 mutant phenocopied the effect of PLK4 inhibition by reducing the number of characteristic visible CS-puncta surrounding the centrosome. Conversely, introduction of a phospho-mimicking PCM1 mutant resulted in larger than normal CS-puncta/aggregates, that clustered tightly around the centrosome in a non-motile manner. This phenotype is by the way highly reminiscent of that reported for SSX2IP knockdown, where CS become trapped at the microtubule minus ends [55,56]. It is plausible that PLK4-mediated reversible phosphorylation of PCM1, promoting PCM1 oligomerization, could represent a mechanism for the re-assembly of CS structures upon completion of mitosis [29].

CS integrity is sensitive to cell cycle changes as well as various intra- and extracellular cues. Perhaps the most dramatic example of this is the rapid cell stress-induced and MAP kinase dependent depletion of CS [19]. This response is mediated by the p38-activated kinase MK2 that phosphorylates CEP131 on Ser-47 and Ser-78 to generate binding sites for 14-3-3 proteins [30]. In human cells, 14-3-3s comprise a family of 7 proteins that form homo- and heterodimers and bind phosphorylated epitopes on target proteins [57]. Binding of 14-3-3 to CEP131 blocks de novo formation of CS without interfering with their dissolution, leading to a gradual decline of microscopically visible CS-puncta. Stress-induced CS depletion has been demonstrated for a number of central CS-markers (e.g., PCM1, CEP131, SSX2IP and CEP290) [30], however, it is entirely possible that subpopulations of CS that are void of these specific markers persist during cellular stress responses. Of note, OFD1-positive CS-like puncta could still be observed in UV-irradiated cells, potentially highlighting the existence of compositionally heterogeneous populations of CS [19]. A couple of reports have described how persistent DNA-damage leads to the nucleation of CS-reminiscent centrin-containing granules [33,58]. UV-induced CS dispersal appears to constitute a more general and acute cell stress response that is induced by a range of p38-activating insults, including the ribotoxic stress agents anisomycin and cycloheximide. The p38-MK2 pathway can also be activated through receptor mediated signalling, for example upon immune stimulation by pro-inflammatory cytokines or bacterial lipopolysaccharides (LPS), and this signalling is required for the establishment of a proper immune response [59]. A recent report uncovered that the centrosome undergoes p38- and JNK-dependent maturation upon exposure of cells to pro-inflammatory signals, an effect that was independent of PLK1 activity and occurred in interphase cells [60]. Activation of these MAP kinases led to increased γ-tubulin and pericentrin at the centrosome and increased microtubule nucleation, and these processes positively impacted on the secretion of pro-inflammatory cytokines (e.g., IL-6 and IL-10). In the absence of a mechanistic explanation for how p38 and JNK activity promote centrosome maturation, it is tempting to speculate that CS remodelling plays a supporting or even critical role during this process. Although it has not been vigorously tested, formation of CS is likely blocked due to p38-MK2-14-3-3 signalling during prolonged immune activation. Subsets of CS associated proteins are either positive or negative regulators of centriole/centrosome duplication and maturation. Potentially, p38-mediated CS disassembly could either make positive regulators available to the centrosome or prevent transport of negative regulators to this structure. As these data potentially place CS dynamics and the centrosome at the crossroads of immune responses, it would be of considerable interest to investigate whether p38-mediated centrosome maturation and CS depletion are functionally linked.

### 1.5. Future Perspectives for the Centriolar Satellite Field

Aided by large-scale genetic screens and proteomic investigations, the last couple of years have witnessed a dramatic expansion in the established inventory of proteins that localize to or communicate with CS [8,28,61]. A substantial number of these factors are found in at least two separate pools, one that is associated directly with the centrosome, and one that resides in CS. A recurring complication in many published studies of CS factors is the fact that it is technically challenging to uncouple their centrosome-specific functions from those directly linked to CS-specific. With some notable differences, such as the case of OFD1, it seems entirely possible that the dual localization of these factors does not have important functional implications but rather represent the function of CS as a key transit point for protein transport to the centrosome. This notion is well in line with the overall perception that the main role of CS is to support the centrosome in its various canonical and specialized tasks, such as organising the microtubule network [4,5], promoting ciliogenesis [5,8,14,15], mitosis [5,62,63], neurogenesis [37,41,64] and potentially even cytokine production [60] (Figure 2B). Judging by their molecular composition it is clear that, depending on cellular demand (e.g., cell-cycle status, energy levels or injury/stress), these CS are capable of both positive and negative regulation of these centrosome-associated processes. Thus, further understanding of how CS are dynamically regulated with regard to subcellular localization, asymmetrical association between mother and daughter centriole, molecular composition and structural integrity, and translating this to the status and function of the centrosome would be of tremendous value in moving the field forward. While such objectives might present considerable technical challenges, the recent identification of CS regulators such as PLK4, MK2, MIB1 and USP9X allows for interventions specifically at the level of CS. Proteomics-based investigation continues to be a valuable tool for the identification of novel CS-associated proteins. Proximity-based interaction screens even allow for identification of transient protein-protein interactions, for example, between protein kinases and their substrate. Indeed, these screens highlight potential roles for novel DUBs (e.g., CYLD), Ubiqutin E3 ligases (e.g., TRIM9 and WWP2) and kinases (e.g., TTK) as regulators of CS [8,28,48,61].

## 2. Materials and Methods

### 2.1. Plasmids and siRNA

Plasmids pGEX2TK-P-GST-14-3-3ε and pGEX2TK-P-GST-14-3-3ζ were described [65]. siRNA transfections were carried out with Lipofectamine RNAiMAX (Life Technologies, Carlsbad, CA, USA) following the manufacturer’s protocol. siRNA (Eurofins, Luxembourg) target sequences used in this study were as follows: Control (siCTRL; 5′-CGUACGCGGAAUACUUCGA-3′); and SSX2IP (siSSX2IP; 5′-GACAGACAGUUACAAUGUA-3′).

### 2.2. Cell Culture and Reagents

Human osteosarcoma U2OS cells and human keratinocyte HaCaT cells were cultured in DMEM medium supplemented with 10% foetal bovine serum, l-glutamine, penicillin and streptomycin. hTERT immortalized human retinal pigmented epithelial (RPE-1) cells were cultured in 50% DMEM and 50% F12 medium with the same supplements as above. All cells were cultured in a humidified 5% CO_2_ incubator at 37 °C. Reagents used were MK2 inhibitor PF3644022 (10 mM, Sigma, Saint Louis, MO, USA), Nocodazole (0.5 µg/mL, Sigma), Taxol (10 µM, Sigma), Anisomycin (1 µg mL^−1^, Cayman Chemical, Ann Arbor, MI, USA), Interleukin-1 beta (2 ng mL^−1^, Preprotech, Rocky Hill, NJ, USA), 4NQO (1 µg mL^−1^, Acros Organics, Morris Plains, NJ, USA), Sorbitol (500 mM, Sigma), Sodium Chloride (500 mM, Sigma), Arsenite (0.5 mM, Sigma) and hydrogen peroxide (5 mM, Merck, Kenilworth, NJ, USA). GST-tagged 14-3-3ε and 14-3-3ζ were purified from *E. coli* and mixed in a 1:1 ratio for use in GST pull-down experiments as described by Blasius et al. [65].

### 2.3. Immunochemical Methods

GST pull-down was performed with Glutathione Sepharose beads (GE healthcare, Chalffint, UK) and carried out in low-salt EBC lysis buffer (150 mM NaCl; 50 mM Tris, pH 7.5; 1 mM EDTA; 0.5% NP40). Antibodies included in this study: CEP131 (A301-425A, Bethyl, WB 1:1000; ab84864, Abcam, Cambridge, UK, IF 1:300), PCM1 (sc-50164, Santa Cruz, IF 1:200), SSX2IP (HPA027306, Sigma-Aldrich, WB 1:1000), MK2 (3042S, Cell Signalling, Danvers, MA, USA, WB 1:1000), HSP27 pS82 (9709, Cell Signalling, WB 1:5000), GST (G7781, Sigma-Aldrich, WB 1:1000), Pericentrin (ab4448, Abcam, IF 1:1000), p38 MAPK (9212S, Cell Signalling, WB 1:1000), p38 MAPK phospho-Thr180/Tyr182 (9216S, Cell Signalling, WB 1:1000), γ-tubulin (T5326, Sigma-Aldrich, IF 1:250), p150 (610473, BD Biosciences, WB 1:10,000), NOGO-B (previously described by Rousseau et al. [66] WB 1:1000), SRF phospho-Ser103 (4261, Cell Signalling, WB 1:1000), and α-tubulin (T9026, Sigma-Aldrich, IF 1:500).

### 2.4. Immunofluorescence Staining and Microscopy

For IF staining of centriolar satellites in combination with γ-tubulin, cells were fixed in ice-cold 1:1 methanol/acetone, dried for 15 min at room temperature (RT) and rehydrated in PBS. Coverslips were incubated with primary antibodies diluted in DMEM for 1 h at RT. Subsequently, coverslips were stained with secondary antibodies (Alexa Fluor 488 and 568, Life Technologies) for 30 min. Finally, coverslips were mounted with Vectashield mounting medium (Vector Laboratories, Burlingame, CA, USA) containing nuclear stain 4,6-diamidino-2-phenylindole (DAPI). For fixation of IF samples of centriolar satellites without γ-tubulin, cells were fixed in 4% formaldehyde (VWR), permeabilized with PBS containing 0.2% Triton X-100 for 5 min and immunostained as above. For live cell imaging, U2OS: Flp-In T-Rex GFP-CEP131 cells [5] were grown in 2-well chambered borosilicate coverglass dishes (Lab-Tek). Immediately before imaging, culture medium was changed to Leibovitz medium (Life Technologies) supplemented with 10% foetal bovine serum (Hyclone) and Penicillin/Streptomycin (Life Technologies). Images and time-lapse movie streams were acquired with an LSM 780 confocal microscope (Carl Zeiss Microimaging Inc.) mounted on a Zeiss-Axiovert 100 M equipped with a Plan-Apochromatic × 63/1.4 oil immersion objective, or a Leica DM4B wide-field microscope (Leica Microsystems) equipped with an HC Plan-Apochromatic × 63/1.3 oil immersion objective. Image acquisition and analysis were carried out with ZEN2010 (Zeiss) and LAS X Software (Leica), respectively. Presence of centriolar satellites was quantified by assessing individual cells for presence of a cluster of PCM1 or CEP131 in direct vicinity of the centrosome in both axial (*x*, *y*) and lateral (*z*) directions. Statistical analysis was carried out using one-way ANOVA with Dunnett’s multiple comparisons test.

### 2.5. Structured Illumination Microscopy

IF fixation and staining was carried out as above, except for 1-min pre-extraction with PBS containing 0.01% Triton X-100, a post-fixation blocking in 2% BSA in PBS for 30 min, and secondary antibody incubation with DAPI (1 µg mL^−1^) before mounting in Vectashield mounting medium (H-1000, Vector Laboratories). Super resolution 3D-SIM images were acquired with Zeiss ELYRA PS.1 Super Resolution Microscope equipped with a Plan-Apochromatic × 63/1.4 oil objective and 405, 488 and 561 HR Diode lasers (50, 200 and 200 mW) with BP420-480/LP750, BP495-575/LP750 and BP570-650/LP750 filters using 1.518 Immersol immersion oil (Carl Zeiss). 15 (512 × 512 pixel) images were taken per z-stack (3 angles and 5 phases) with a typical z-stack height of 6 µM with a z-stack difference of 90 nm. Following acquisition, images were reconstructed using Zeiss ZEN Black 2012 software’s algorithm for structured illumination with manual processing (‘Auto Noise Filter’, ‘shifted baseline’, ‘100;83;83 sectioning’ and ‘theoretical PSF’ settings). To correct for spatial misalignments, images were channel aligned using a matrix calibration based on stained beads which was calculated by the Zeiss ZEN Black 2012 software using the ‘Affine’ transformation. Lateral resolution was estimated to 125 nm and quality of raw data was validated, both using SimCheck (Fiji plugin, ImageJ). All 3D-SIM images are shown as 3D projections visualized by Zeiss ZEN Black 2012 3D module using the transparent mode.

### 2.6. Analysis of Centriolar Satellite Trajectories

U2OS: Flp-In T-Rex GFP-CEP131 [30] cells were left untreated or incubated with 500 mM sorbitol prior to live cell imaging. Movie streams were acquired at a rate of 1 frame per 8 s (100 images per movie). Particle tracking analysis was carried out using the MOSAIC group ImageJ/Fiji plugin [67] with the following settings; Radius: 5, Cutoff: 0, Percentile: 0.18, Link Range: 5, Displacement 15, Dynamics: Brownian motion, and default advanced options. Briefly, defined particle positions were detected in each frame of the movie stream and then linked forming individual trajectories. The trajectories were then processed and plotted using R version 3.4.0. Statistical analysis was carried out using Wilcoxon rank sum test with continuity correction.

## 3. Results

### 3.1. Osmotic Stress Counteracts p38-Mediated Dissolution of Centriolar Satellites

**Figure 3:**
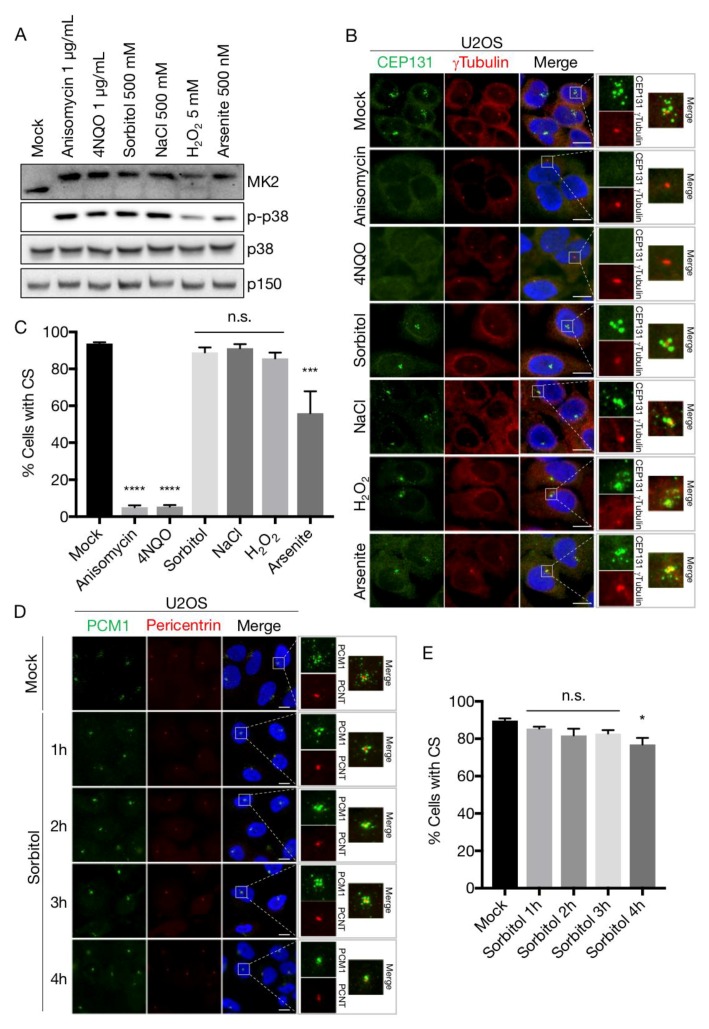
Hyperosmotic conditions protect centriolar satellites from p38-mediated depletion. (**A**) U2OS cells were treated with the indicated stress agents (1 h) and lysates were analysed by immunoblotting with antibodies against phospho-p38, total p38, MK2 and p150 (loading control). (**B**) Cells treated as in (**A**) were immunostained with antibodies against CEP131 and γ-tubulin and counterstained for nuclear content with DAPI. (**C**) Quantification of (**B**). At least 100 cells were scored per condition in 3 independent experiments. Bars indicate the mean +/− SEM. *p*-values were calculated from a one-way ANOVA using Dunnett’s correction for multiple testing. (**D**) U2OS cells were incubated in the presence of 500 mM sorbitol for the indicated times. Cells were immunostained with antibodies against PCM1 and pericentrin and counterstained for nuclear content with DAPI. (**E**) Quantification of (**D**) performed as in (**C**). *, *p* < 0.05; ***, *p* < 0.0005; ****, *p* < 0.0001, n.s.; not significant. All scale bars, 10 μm. CS; centriolar satellites.

We previously uncovered the mechanism of cellular stress-induced dissolution of CS [19,30]. Briefly, the MK2 kinase, upon activation by p38, phosphorylates Ser-47 and Ser-78 of CEP131 to establish an affinity platform for 14-3-3 proteins. The ensuing binding reaction blocks formation of new satellites and, due to the gradual dissolution of existing structures, results in the rapid exhaustion of CS. This response is readily observed upon a vast array of p38 activating insults, most notably UV-irradiation and the ribotoxic stress agents anisomycin and cycloheximide, and even occurs upon expression of an active form of the p38-activating kinase MKK6. In an attempt to generalize these findings, we challenged U2OS cells with a wide array of p38-activating stress agents including NaCl and sorbitol (osmotic stress), arsenite and H_2_O_2_ (oxidative stress), anisomycin (ribotoxic stress) and the UV-mimetic compound 4NQO. All of these treatments led to robust and fast (1 h) appearance of the phosphorylated and activated forms of the p38 and MK2 kinases (Figure 3A). Surprisingly, however, osmotic and to some degree oxidative stress did not result in the loss of CS as judged by widefield fluorescence microscopy of CEP131 and PCM1 immunostainings (Figure 3B–D). Even at prolonged times (up to 4 h) after sorbitol addition the satellites remained relatively intact (Figure 3D,E). This was in contrast to the complete CS depletion observed after stressing cells with 4NQO or anisomycin (Figure 3B,C). Both of these agents impair protein synthesis, raising the possibility that only ribotoxic stress-induced p38 activation constitutes the cellular signal that cause exhaustion of CS. However, incubation with IL-1β, a pro-inflammatory cytokine that activates p38 in U2OS cells [68], was also accompanied by a near complete yet transient depletion of CS (Supplementary Materials Figure S1A,B). The transiency of the response is likely to be the result of p38-controlled transcription of MAP kinase phosphatases of the DUSP family [69]. This negative feedback mechanism requires protein translation and thus does not operate in anisomycin-treated cells. Indeed, the phosphorylation of both p38 and MK2 was already on the decline 90 min after IL-1β treatment (Supplementary Materials Figure S1C). Our results indicate that a diverse repertoire of p38 activating insults is accompanied by loss of centriolar satellites and that the persistence of these structures under osmotic and oxidative stress conditions represent unique cases.

### 3.2. Sorbitol Does Not Interfere with p38-MK2 Signalling

**Figure 4:**
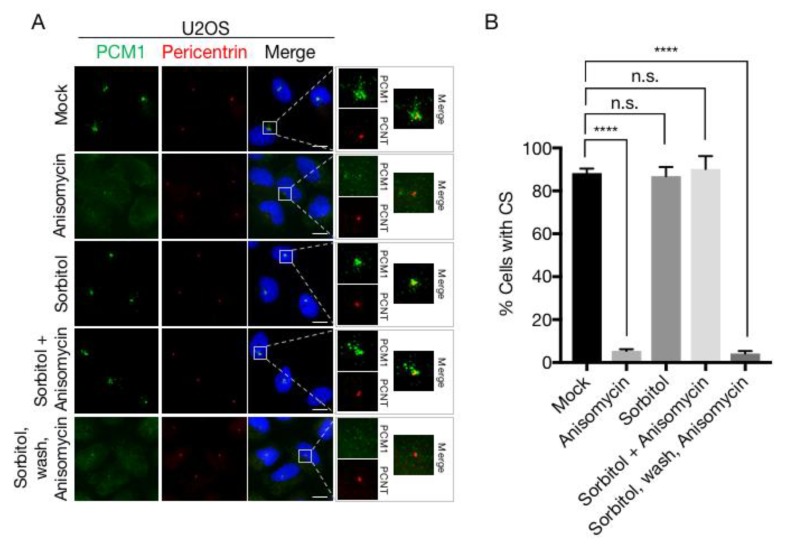

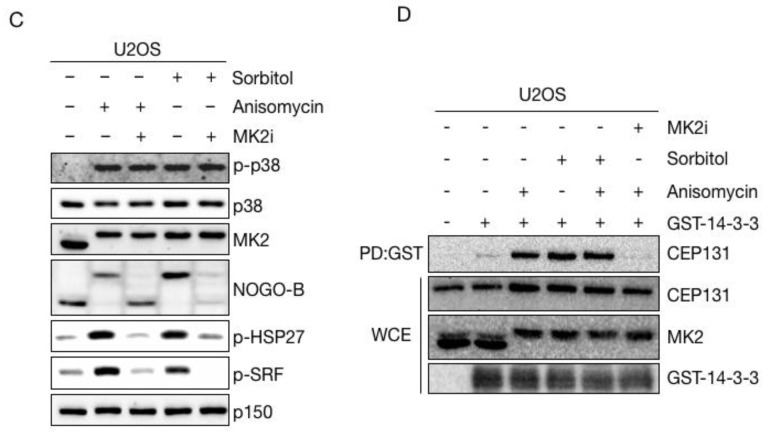
Sorbitol does not interfere with phosphorylation of MK2 targets. (**A**) U2OS cells were treated with anisomycin or sorbitol (1 h) in the indicated combinations. Cells were immunostained with antibodies against CEP131 and counterstained for nuclear content with DAPI. PCNT; Pericentrin. Scale bars, 10 μm. (**B**) Quantification of (**A**). At least 100 cells were scored per condition in 3 independent experiments. Bars indicate the mean +/− SEM. *p*-values were calculated from a one-way ANOVA using Dunnett’s correction for multiple testing. ****, *p* < 0.0001, n.s.; not significant. CS; centriolar satellites. (**C**) U2OS cells were pretreated with an MK2 inhibitor (MK2i—1 h) and incubated in the presence of sorbitol and anisomycin as indicated. Lysates were analysed by immunoblotting with antibodies against phospho-p38, total p38, MK2, NOGO-B, phospho-HSP27, phospho-SRF and p150 (loading control). (**D**) Lysates from cells treated as in (c) were incubated with recombinant GST or GST-14-3-3 and subjected to GST pulldown (PD). Pulldown material and whole cell extracts (WCE) were analysed by immunoblotting with antibodies against CEP131, MK2 and GST.

To explain our surprising finding that robust activation of p38 by osmotic stress agents is not accompanied by CS depletion, we considered the possibility that CS loss requires an additional signalling pathway that is not activated after osmotic stress. However, apart from leaving the structures intact, treatment with sorbitol or NaCl also protected CS from the destabilizing effects of ansiomycin (Figure 4A,B and Figure S2A,B), an effect which we also observed in a diploid fibroblast cell line (RPE-1) and a keratinocyte cell line (HaCaT) (Figure S2C-E). We also noted that the protective effect of hyperosmolarity on CS was completely reversible, as removal of sorbitol or NaCl from the medium and the immediate exposure of cells to anisomycin lead to rapid and complete CS loss (Figure 4A,B and Figure S2A,B).

Next, we entertained the possibility that osmotic stress, even though it causes phosphorylation of both p38 and MK2 (Figure 3A), could interfere with the activity of MK2 and the ability of this kinase to phosphorylate its physiological substrates. However, sorbitol induced robust MK2-dependent phosphorylation of a number of well-studied substrates, including HSP27, NOGO-B and SRF in all the cell lines that we tested (Figure 4C and Figure S2F). Also, MK2-dependent phosphorylation and ensuing 14-3-3 binding of CEP131, the reaction that normally underlies CS depletion, was readily observed in sorbitol-treated cells (Figure 4D). These experiments indicate that even though sorbitol inhibits p38-mediated CS exhaustion, it does not impair any of the known signalling events required for the reaction.

### 3.3. High Osmolarity Renders CS Immobile and Insensitive to Disassembly and Redistribution

**Figure 5:**
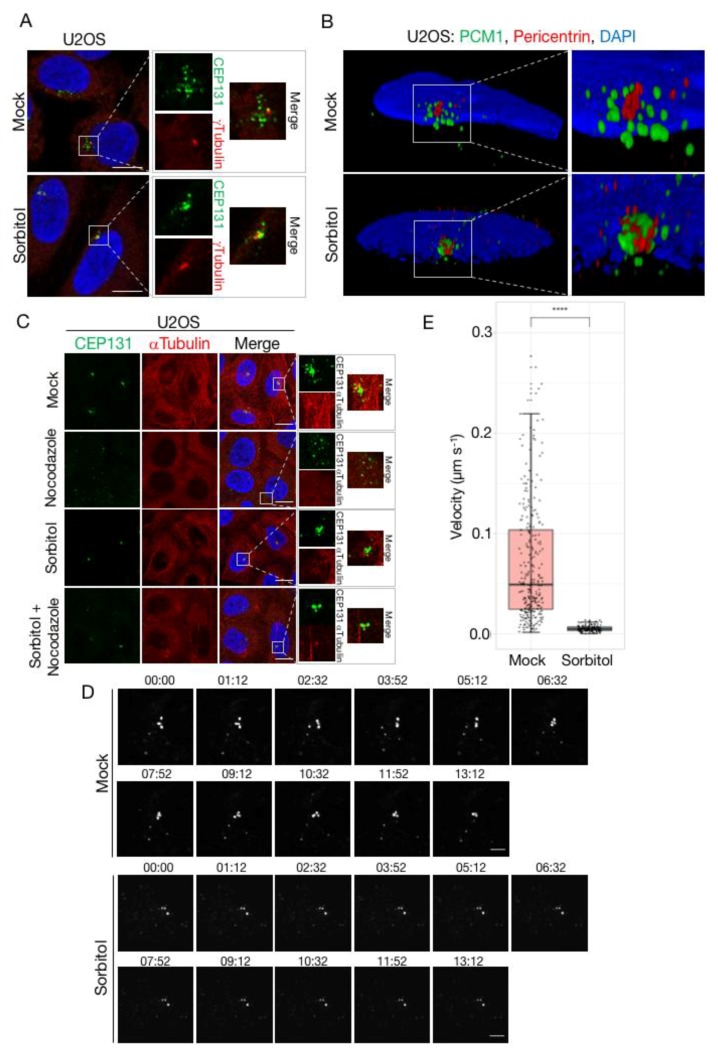
Dispersal and mobility of centriolar satellites is impaired by sorbitol. (**A**) U2OS cells were left untreated (mock) or incubated in the presence of sorbitol (1 h). Cells were immunostained with antibodies against CEP131 and γ-tubulin, counterstained for nuclear content with DAPI, and subjected to confocal microscopy. Scale bars, 10 μm. (**B**) Cells from (**A**) were immunostained with antibodies against PCM1 and pericentrin, counterstained for nuclear content with DAPI, and subjected to 3D-SIM microscopy. Pictures represent reconstructed images shown as 3D projections. (**C**) U2OS cells were pre-treated with sorbitol (1 h) and incubated in the presence of nocodazole (3 h) as indicated. Cells were immunostained with antibodies against CEP131 and α-tubulin and counterstained for nuclear content with DAPI. Scale bars, 10 μm. (**D**) U2OS cells stably expressing low levels of GFP-CEP131 were incubated in the presence or absence of sorbitol and subjected to time-lapse microscopy. Note the high mobility of satellites in mock-treated cells compared to their near-complete immobility in sorbitol-treated cells (see also Supplementary movies 1 and 2). Scale bars, 5 μm. (**E**) Quantification of satellite velocities from (**D**). Trajectories of individual satellites were identified using the MOSAIC ImageJ/Fiji plug-in. Trajectories from three independent movie streams were used to calculate velocities of individual satellites. The two groups were compared using Wilcoxon rank sum test with continuity correction. *p* < 2.2 × 10^−16^. (see also Supplementary movies 3 and 4).

As all of the known signalling events underlying stress-induced CS depletion occur normally upon osmotic stress, we argued that the inability of cells to resolve CS under these conditions could be connected to changes to the structure or dynamics of satellites. Applying confocal microscopy to CEP131- and PCM1-stained CS, we noted that the appearance of these structures changed markedly upon treatment of cells with sorbitol (Figure 5A and Figure S3A). Instead of the normal granular and scattered localization in the vicinity of the centrosome, sorbitol treatment was associated with an apparent coalescence of satellites and a decrease in their distance to the centrosome. This observation could indicate a change in the equilibrium between anterograde and retrograde transport of CS and is reminiscent of the phenotype reported for SSX2IP knockdown cells, where the satellites become trapped at the minus ends of microtubules [55]. This mechanism could however not explain the resistance to depletion after osmotic stress, as we readily observed CS exhaustion upon treatment of SSX2IP-deficient cells with anisomycin (Figure S3B–D). We also examined whether the sorbitol-induced concentration of CS directly proximal to the centrosome was associated with an apparent change in their ultrastructure. To this end, we increased imaging resolution by applying 3-Dimensional Structured Illumination Microscopy (3D-SIM) to immunostainings of PCM1 and the centrosomal marker pericentrin. While these experiments corroborated our previous observations regarding the altered spatial organization of CS in sorbitol-treated cells, we did not notice any obvious changes to the appearance of individual CS as judged 3D projection of the PCM1 signal in the structures (Figure 5B).

The proper localization of centriolar satellites are known to be highly dependent on the integrity of the microtubule network. Treatment of U2OS cells with the microtubule depolymerizing agent nocodazole was accompanied by the dispersal but not disassembly of satellites as previously reported [4] (Figure 5C). Despite their altered localization, these dispersed satellites still dissolved completely upon anisomycin-induced p38 activation, suggesting that the dynamic formation and dissolution of CS is not connected to their cellular localization (Figure S3E). Remarkably, sorbitol also blocked the nocodazole-induced CS reorganization, without interfering with nocodazole-induced loss of microtubule organization (Figure 5C). We also treated cells with the microtubule stabilizing agent taxol, resulting in the appearance of highly filamentous microtubuli and near complete depletion of CS (Figure S3F). Also under these experimental conditions, sorbitol protected CS from dissolution without interfering with the effects of taxol on microtubule stabilization (if anything, this effect was exacerbated).

High osmolarity blocks several mechanistically independent processes that lead to the dispersion or depletion of CS. All of these phenomena depend on the dynamic properties of CS, including their rapid microtubule-dependent transport, and the balance between satellite formation and dissolution. These observations prompted us to speculate that sorbitol and other osmotic stress agents could perturb the dynamics of CS. To investigate this, we performed time-lapse microscopy of a previously established cell line stably expressing low levels of GFP-CEP131. Under normal culture conditions, we observed high mobility of CS, including localized exploration by individual CS as well as rapid step-wise translocation (Figure 5D; Video S1). These latter events probably represent microtubule-dependent transport towards or away from the centrosome. We also readily observed formation and dissolution events, although we cannot exclude that some of these represent movement of CS in and out of the focal plane. This highly dynamical behaviour was in stark contrast to cells cultivated in sorbitol containing medium, where all satellites exhibited a highly static behaviour (Figure 5D; Video S2). Thus, we did not observe any of the above-mentioned events, and the very limited movement of individual structures likely represented cellular movements rather than CS movement. Applying a particle tracking approach, we were able to reconstruct trajectories of individual CS and to measure the distances travelled by these structures during the course of our time-lapse experiments. This analysis confirmed that CS are remarkably static in sorbitol-treated cells, in stark contrast to the highly dynamic behaviour observed under normal culture conditions (Figure 5E).

Even though osmotic stress is associated with robust activation of p38 and MK2 and ensuing phosphorylation and 14-3-3 binding of CEP131, it is not accompanied by CS depletion. Our experiments indicate that osmotic stress strongly restricts the normal dynamic behaviour of CS, and interferes with formation and dissolution of these structures.

## 4. Discussion

Centriolar satellites serve as carriers of cargo to the centrosome and as such they play key roles in many processes that impinge on both canonical and specialized functions of the centrosome. Consequently, regulation of CS abundance, integrity and composition are central to modulation of centrosome function in a variety of cellular responses, including ciliogenesis, neurite outgrowth and cell division. The rapid remodelling of CS is aided by their dynamic nature, underscored by their continuous formation and dissolution, and constant translocation along the microtubule network. Activation of the MAP kinase p38 fires a pathway that blocks the formation of CS which, given their dynamic properties, leads to the rapid exhaustion of these cellular structures. In this work, we describe how hyperosmotic conditions interfere with the normal dynamics of CS, rendering them immobile and insensitive to disruption of the microtubule network, and refractory to p38-dependent depletion. Osmotic stress induced by sorbitol and sodium chloride is in itself a potent trigger of p38 signalling, leading to phosphorylation and 14-3-3-dependent sequestration of CEP131 that will block CS formation. However, due to a simultaneous block to the normal turnover of CS, these structures persist in cells exposed to high concentrations of osmolytes.

The molecular mechanisms underpinning the osmotic stress-induced CS immobilization and protection from dissolution remain elusive. It is plausible that the mechanisms at play are entirely passive, and that high cellular osmolarity interferes with the motor function of kinesins and dyneins, resulting in an uncoupling of CS from microtubules. Alternatively, the transient complexes between CS, the targeting factor dynactin and motor proteins could be disturbed under these specific experimental conditions. Such scenarios could explain the lack of CS transport that we observe, but is unlikely to be the underlying cause of the block to dissolution, and so far, CS transport and turnover has not been functionally linked. Of note, we also found that upon forced microtubule depolymerisation by nocodazole treatment, CS depletion still occurred after activation of p38 by anisomycin (Figure S3E). 

Rather than being the result of pleiotropic effects of high osmolarity, CS immobilization and protection from dissolution could also be caused by active signalling mechanisms uniquely induced by osmotic stress. Such parallel signalling could interfere with CS turnover and transport, and potentially explain the static behaviour of these structures that we observe specifically upon osmotic shock. The concentrations of osmolytes used in this study also activate the DNA damage response (DDR) [70] and interferes with many basic cellular functions [71]. In this connection, we were not able to circumvent sorbitol-induced CS protection by inhibition of the major DDR-associated kinases, and UV irradiation which also simultaneously leads to DNA damage and p38 activation is accompanied by robust CS depletion [19]. Besides rendering CS immobile, sorbitol treatment also causes these structures to move into the immediate vicinity of the centrosome. A similar phenomenon was reported in SSX2IP-deficient cells, and was suggested to be the result of tethering of CS to microtubule minus ends [55]. Sorbitol treatment also protects CS from nocodazol induced relocalization, suggesting that microtubules are not involved in our case, but we cannot exclude the possibility that sorbitol induces tethering to another cellular structure, such as the centrosome itself.

Our work highlights some gaps in our understanding of CS biology. Although the dynamical properties of these structure are clearly essential to their function, we do not have any tangible insight into the processes that govern their continuous formation and dissolution. The underlying mechanisms could revolve around an inherent propensity of CS factors to aggregate on the one hand, coupled with a stochastic instability of the assembled CS structure on the other. More likely, formation or dissolution of CS, or even both, could be induced by active signalling events to manifest their dynamic properties. Candidate mechanisms could be p97-mediated and ubiquitin-dependent segregation, autophagy or phosphorylation-induced stabilization and destabilization of the structure. Of note, a number of ubiquitin ligases, kinases and mediators of autophagy reside in CS.

Experimentally induced osmotic stress conditions only have few physiological counterparts in the multicellular mammalian organism. Best studied are cells of the renal medulla that regularly experience high osmolarity connected to the concentration of urine in the kidneys [72]. These physiological conditions cause the periodic activation of MAP kinases including p38, DDR and cell cycle checkpoints, all of which constitute important defence mechanisms to maintain cellular homeostasis in the kidney. Another example of a physiologically relevant osmotic stress condition is chronic dry eye disease resulting from tear hyperosmolarity [72]. Immobilization and protection of CS may also manifest during these osmotic stress responses with unknown functional consequences.

## 5. Conclusions

In conclusion, we have uncovered how osmotic shock renders CS immobile and insensitive to regulatory input. This also makes CS refractory to the p38-mediated depletion associated with a range other stress stimuli and inflammatory signalling. The consequent separation of p38 activation and CS removal is unique to osmotic stress conditions and is likely to have physiological implications in a range of human tissues, which should be the target of future studies.

## Supplementary Materials

The following are available online at http://www.mdpi.com/2073-4409/7/7/65/s1, Figure S1: IL-1β induces transient depletion of centriolar satellites, Figure S2: Sorbitol blocks p38-mediated centriolar satellite depletion in several human cell lines, Figure S3: Effects of sorbitol on centriolar satellite localization, Video S1: Mobility of centriolar satellites in untreated cells, Video S2: Mobility of centriolar satellites in sorbitol-treated cells, Video S3: Trajectories of individual centriolar satellites in untreated cells, Video S4: Trajectories of individual centriolar satellites in sorbitol-treated cells.

## References

[1] Balczon R., Bao L., Zimmer W.E. (1994). PCM-1, A 228-kD centrosome autoantigen with a distinct cell cycle distribution. J. Cell Biol..

[2] Kubo A., Sasaki H., Yuba-Kubo A., Tsukita S., Shiina N. (1999). Centriolar satellites: Molecular characterization, ATP-dependent movement toward centrioles and possible involvement in ciliogenesis. J. Cell Biol..

[3] Kubo A., Tsukita S. (2003). Non-membranous granular organelle consisting of PCM-1: Subcellular distribution and cell-cycle-dependent assembly/disassembly. J. Cell Sci..

[4] Dammermann A., Merdes A. (2002). Assembly of centrosomal proteins and microtubule organization depends on PCM-1. J. Cell Biol..

[5] Tollenaere M.A.X., Mailand N., Bekker-Jensen S. (2015). Centriolarsatellites: Key mediators of centrosome functions. Cell. Mol. Life Sci..

[6] Hori A., Toda T. (2017). Regulation of centriolar satellite integrity and its physiology. Cell. Mol. Life Sci..

[7] Bärenz F., Mayilo D., Gruss O.J. (2011). Centriolar satellites: Busy orbits around the centrosome. Eur. J. Cell Biol..

[8] Gupta G.D., Coyaud É., Gonçalves J., Mojarad B.A., Liu Y., Wu Q., Gheiratmand L., Comartin D., Tkach J.M., Cheung S.W.T. (2015). A Dynamic Protein Interaction Landscape of the Human Centrosome-Cilium Interface. Cell.

[9] Kim J.C., Badano J.L., Sibold S., Esmail M.A., Hill J., Hoskins B.E., Leitch C.C., Venner K., Ansley S.J., Ross A.J. (2004). The Bardet-Biedl protein BBS4 targets cargo to the pericentriolar region and is required for microtubule anchoring and cell cycle progression. Nat. Genet..

[10] Kodani A., Tonthat V., Wu B., Sütterlin C. (2010). Par6 alpha interacts with the dynactin subunit p150 Glued and is a critical regulator of centrosomal protein recruitment. Mol. Biol. Cell.

[11] Kim J., Krishnaswami S.R., Gleeson J.G. (2008). CEP290 interacts with the centriolar satellite component PCM-1 and is required for Rab8 localization to the primary cilium. Hum. Mol. Genet..

[12] Nicolas L., Merdes A. (2018). Centriolar satellites prevent uncontrolled degradation of centrosome proteins: A speculative review. Cell Stress.

[13] Reiter J.F., Leroux M.R. (2017). Genes and molecular pathways underpinning ciliopathies. Nat. Rev. Mol. Cell Biol..

[14] Lopes C.A., Prosser S.L., Romio L., Hirst R.A., O’Callaghan C., Woolf A.S., Fry A.M. (2011). Centriolar satellites are assembly points for proteins implicated in human ciliopathies, including oral-facial-digital syndrome 1. J. Cell Sci..

[15] Chamling X., Seo S., Searby C.C., Kim G., Slusarski D.C., Sheffield V.C. (2014). The Centriolar Satellite Protein AZI1 Interacts with BBS4 and Regulates Ciliary Trafficking of the BBSome. PLoS Genet..

[16] Zhang Y., Seo S., Bhattarai S., Bugge K., Searby C.C., Zhang Q., Drack A.V., Stone E.M., Sheffield V.C. (2014). BBS mutations modify phenotypic expression of CEP290-related ciliopathies. Hum. Mol. Genet..

[17] Kodani A., Yu T.W., Johnson J.R., Jayaraman D., Johnson T.L., Al-Gazali L., Sztriha L., Partlow J.N., Kim H., Krup A.L. (2015). Centriolar satellites assemble centrosomal microcephaly proteins to recruit CDK2 and promote centriole duplication. eLife.

[18] Tang Z., Lin M.G., Stowe T.R., Chen S., Zhu M., Stearns T., Franco B., Zhong Q. (2013). Autophagy promotes primary ciliogenesis by removing OFD1 from centriolar satellites. Nature.

[19] Villumsen B.H., Danielsen J.R., Povlsen L., Sylvestersen K.B., Merdes A., Beli P., Yang Y.-G., Choudhary C., Nielsen M.L., Mailand N. (2013). A new cellular stress response that triggers centriolar satellite reorganization and ciliogenesis. EMBO J..

[20] De Saram P., Iqbal A., Murdoch J.N., Wilkinson C.J. (2017). BCAP is a centriolar satellite protein and inhibitor of ciliogenesis. J. Cell Sci..

[21] Kamiya A., Tan P.L., Kubo K.-I., Engelhard C., Ishizuka K., Kubo A., Tsukita S., Pulver A.E., Nakajima K., Cascella N.G. (2008). Recruitment of PCM1 to the centrosome by the cooperative action of DISC1 and BBS4: A candidate for psychiatric illnesses. Arch. Gen. Psychiatry.

[22] Nachury M.V., Loktev A.V., Zhang Q., Westlake C.J., Peränen J., Merdes A., Slusarski D.C., Scheller R.H., Bazan J.F., Sheffield V.C. (2007). A core complex of BBS proteins cooperates with the GTPase Rab8 to promote ciliary membrane biogenesis. Cell.

[23] Stowe T.R., Wilkinson C.J., Iqbal A., Stearns T. (2012). The centriolar satellite proteins Cep72 and Cep290 interact and are required for recruitment of BBS proteins to the cilium. Mol. Biol. Cell.

[24] Lacey K.R., Jackson P.K., Stearns T. (1999). Cyclin-dependent kinase control of centrosome duplication. Proc. Natl. Acad. Sci. USA.

[25] Rajagopalan H., Lengauer C. (2004). Aneuploidy and cancer. Nature.

[26] Sansregret L., Swanton C. (2017). The Role of Aneuploidy in Cancer Evolution. Cold Spring Harb. Perspect. Med..

[27] Li X., Song N., Liu L., Liu X., Ding X., Song X., Yang S., Shan L., Zhou X., Su D. (2017). USP9X regulates centrosome duplication and promotes breast carcinogenesis. Nat. Commun..

[28] Firat-Karalar E.N., Rauniyar N., Yates J.R., Stearns T. (2014). Proximity Interactions among Centrosome Components Identify Regulators of Centriole Duplication. Curr. Biol..

[29] Hori A., Barnouin K., Snijders A.P., Toda T. (2016). A non-canonical function of Plk4 in centriolar satellite integrity and ciliogenesis through PCM1 phosphorylation. EMBO Rep..

[30] Tollenaere M.A.X., Villumsen B.H., Blasius M., Nielsen J.C., Wagner S.A., Bartek J., Beli P., Mailand N., Bekker-Jensen S. (2015). P38- and MK2-dependent signalling promotes stress-induced centriolar satellite remodelling via 14-3-3-dependent sequestration of CEP131/AZI1. Nat. Commun..

[31] Wang G., Chen Q., Zhang X., Zhang B., Zhuo X., Liu J., Jiang Q., Zhang C. (2013). PCM1 recruits Plk1 to the pericentriolar matrix to promote primary cilia disassembly before mitotic entry. J. Cell Sci..

[32] Shearer R.F., Frikstad K.M., McKenna J., McCloy R.A., Deng N., Burgess A., Stokke T., Patzke S., Saunders D.N. (2018). The E3 ubiquitin ligase UBR5 regulates centriolar satellite stability and primary cilia. Mol. Biol. Cell.

[33] Löffler H., Fechter A., Liu F.Y., Poppelreuther S., Krämer A. (2012). DNA damage-induced centrosome amplification occurs via excessive formation of centriolar satellites. Oncogene.

[34] Joachim J., Razi M., Judith D., Wirth M., Calamita E., Encheva V., Dynlacht B.D., Snijders A.P., O’Reilly N., Jefferies H.B.J. (2017). Centriolar Satellites Control GABARAP Ubiquitination and GABARAP-Mediated Autophagy. Curr. Biol..

[35] Behrends C., Sowa M.E., Gygi S.P., Harper J.W. (2010). Network organization of the human autophagy system. Nature.

[36] Joachim J., Tooze S.A. (2017). Centrosome to autophagosome signaling: Specific GABARAP regulation by centriolar satellites. Autophagy.

[37] Tozer S., Baek C., Fischer E., Goiame R., Morin X. (2017). Differential Routing of Mindbomb1 via Centriolar Satellites Regulates Asymmetric Divisions of Neural Progenitors. Neuron.

[38] Pierfelice T., Alberi L., Gaiano N. (2011). Notch in the vertebrate nervous system: An old dog with new tricks. Neuron.

[39] Engelender S., Sharp A.H., Colomer V., Tokito M.K., Lanahan A., Worley P., Holzbaur E.L., Ross C.A. (1997). Huntingtin-associated protein 1 (HAP1) interacts with the p150Glued subunit of dynactin. Hum. Mol. Genet..

[40] Keryer G., Pineda J.R., Liot G., Kim J., Dietrich P., Benstaali C., Smith K., Cordelières F.P., Spassky N., Ferrante R.J. (2011). Ciliogenesis is regulated by a huntingtin-HAP1-PCM1 pathway and is altered in Huntington disease. J. Clin. Investig..

[41] Puram S.V., Kim A.H., Ikeuchi Y., Wilson-Grady J.T., Merdes A., Gygi S.P., Bonni A. (2011). A CaMKIIβ signaling pathway at the centrosome regulates dendrite patterning in the brain. Nat. Neurosci..

[42] Pampliega O., Orhon I., Patel B., Sridhar S., Díaz-Carretero A., Beau I., Codogno P., Satir B.H., Satir P., Cuervo A.M. (2013). Functional interaction between autophagy and ciliogenesis. Nature.

[43] Dikic I., Elazar Z. (2018). Mechanism and medical implications of mammalian autophagy. Nat. Rev. Mol. Cell Biol..

[44] Wang L., Lee K., Malonis R., Sanchez I., Dynlacht B.D. (2016). Tethering of an E3 ligase by PCM1 regulates the abundance of centrosomal KIAA0586/Talpid3 and promotes ciliogenesis. eLife.

[45] Singla V., Romaguera-Ros M., Garcia-Verdugo J.M., Reiter J.F. (2010). Ofd1, a human disease gene, regulates the length and distal structure of centrioles. Dev. Cell.

[46] Ferrante M.I., Zullo A., Barra A., Bimonte S., Messaddeq N., Studer M., Dollé P., Franco B. (2006). Oral-facial-digital type I protein is required for primary cilia formation and left-right axis specification. Nat. Genet..

[47] Coene K.L.M., Roepman R., Doherty D., Afroze B., Kroes H.Y., Letteboer S.J.F., Ngu L.H., Budny B., van Wijk E., Gorden N.T. (2009). OFD1 is mutated in X-linked Joubert syndrome and interacts with LCA5-encoded lebercilin. Am. J. Hum. Genet..

[48] Akimov V., Rigbolt K.T.G., Nielsen M.M., Blagoev B. (2011). Characterization of ubiquitination dependent dynamics in growth factor receptor signaling by quantitative proteomics. Mol. Biosyst..

[49] Wang Q., Tang Y., Xu Y., Xu S., Jiang Y., Dong Q., Zhou Y., Ge W. (2017). The X-linked deubiquitinase USP9X is an integral component of centrosome. J. Biol. Chem..

[50] Cajanek L., Glatter T., Nigg E.A. (2015). The E3 ubiquitin ligase Mib1 regulates Plk4 and centriole biogenesis. J. Cell Sci..

[51] Hames R.S., Crookes R.E., Straatman K.R., Merdes A., Hayes M.J., Faragher A.J., Fry A.M. (2005). Dynamic recruitment of Nek2 kinase to the centrosome involves microtubules, PCM-1, and localized proteasomal degradation. Mol. Biol. Cell.

[52] Spalluto C., Wilson D.I., Hearn T. (2013). Evidence for centriolar satellite localization of CDK1 and cyclin B2. Cell Cycle.

[53] Avidor-Reiss T., Gopalakrishnan J. (2013). Building a centriole. Curr. Opin. Cell Biol..

[54] Habedanck R., Stierhof Y.-D., Wilkinson C.J., Nigg E.A. (2005). The Polo kinase Plk4 functions in centriole duplication. Nat. Cell Biol..

[55] Hori A., Ikebe C., Tada M., Toda T. (2014). Msd1/SSX2IP-dependent microtubule anchorage ensures spindle orientation and primary cilia formation. EMBO Rep..

[56] Bärenz F., Inoue D., Yokoyama H., Tegha-Dunghu J., Freiss S., Draeger S., Mayilo D., Cado I., Merker S., Klinger M. (2013). The centriolar satellite protein SSX2IP promotes centrosome maturation. J. Cell Biol..

[57] Fu H., Subramanian R.R., Masters S.C. (2000). 14-3-3 proteins: Structure, function, and regulation. Annu. Rev. Pharmacol. Toxicol..

[58] Prosser S.L., Straatman K.R., Fry A.M. (2009). Molecular dissection of the centrosome overduplication pathway in S-phase-arrested cells. Mol. Cell. Biol..

[59] Cuadrado A., Nebreda A.R. (2010). Mechanisms and functions of p38 MAPK signalling. Biochem. J..

[60] Vertii A., Ivshina M., Zimmerman W., Hehnly H., Kant S., Doxsey S. (2016). The Centrosome Undergoes Plk1-Independent Interphase Maturation during Inflammation and Mediates Cytokine Release. Dev. Cell.

[61] Andersen J.S., Wilkinson C.J., Mayor T., Mortensen P., Nigg E.A., Mann M. (2003). Proteomic characterization of the human centrosome by protein correlation profiling. Nature.

[62] Kim K., Rhee K. (2011). The pericentriolar satellite protein CEP90 is crucial for integrity of the mitotic spindle pole. J. Cell Sci..

[63] Oshimori N., Li X., Ohsugi M., Yamamoto T. (2009). Cep72 regulates the localization of key centrosomal proteins and proper bipolar spindle formation. EMBO J..

[64] Kwon D.Y., Dimitriadi M., Terzic B., Cable C., Hart A.C., Chitnis A., Fischbeck K.H., Burnett B.G. (2013). The E3 ubiquitin ligase mind bomb 1 ubiquitinates and promotes the degradation of survival of motor neuron protein. Mol. Biol. Cell.

[65] Blasius M., Wagner S.A., Choudhary C., Bartek J., Jackson S.P. (2014). A quantitative 14-3-3 interaction screen connects the nuclear exosome targeting complex to the DNA damage response. Genes Dev..

[66] Rousseau S., Peggie M., Campbell D.G., Nebreda A.R., Cohen P. (2005). Nogo-B is a new physiological substrate for MAPKAP-K2. Biochem. J..

[67] Sbalzarini I.F., Koumoutsakos P. (2005). Feature point tracking and trajectory analysis for video imaging in cell biology. J. Struct. Biol..

[68] Kulawik A., Engesser R., Ehlting C., Raue A., Albrecht U., Hahn B., Lehmann W.D., Gaestel M., Klingmuller U., Haussinger D. (2017). IL-1beta-induced and p38(MAPK)-dependent activation of the mitogen-activated protein kinase-activated protein kinase 2 (MK2) in hepatocytes: Signal transduction with robust and concentration-independent signal amplification. J. Biol. Chem..

[69] Lang R., Hammer M., Mages J. (2006). DUSP meet immunology: Dual specificity MAPK phosphatases in control of the inflammatory response. J. Immunol..

[70] Kultz D., Chakravarty D. (2001). Hyperosmolality in the form of elevated NaCl but not urea causes DNA damage in murine kidney cells. Proc. Natl. Acad. Sci. USA.

[71] Finan J.D., Guilak F. (2010). The effects of osmotic stress on the structure and function of the cell nucleus. J. Cell. Biochem..

[72] Brocker C., Thompson D.C., Vasiliou V. (2012). The role of hyperosmotic stress in inflammation and disease. Biomol. Concepts.

