# Supplementary material for: Osmotic Stress Blocks Mobility and Dynamic Regulation of Centriolar Satellites
Source: Cells. 2018 Jun 22;7(7):65. doi: 10.3390/cells7070065 (PMC6070812; doi:10.3390/cells7070065)
Supplement: Supplementary file 1 [file cells-07-00065-s001.zip › Supplemental figures.pdf]

Nielsen et al Supplementary Materials

Figure S1.

IL-1β induces transient depletion of centriolar satellites

- **a.** U2OS cells were treated with IL-1β (left) or anisomycin (right) for the indicated times. Cells were immunostained with antibodies against PCM1 and pericentrin and counterstained for nuclear content with DAPI. PCNT; Pericentrin. Scale bars, 10 μm.
- **b.** Quantification of (a). At least 100 cells were scored per condition in 3 independent experiments. Bars indicate the mean +/- SEM. CS; centriolar satellites.
- **c.** Lysates of cells from (a) were analysed by immunoblotting with antibodies against phospho-p38, total p38, MK2 and p150 (loading control).

Nielsen et al, Figure S1

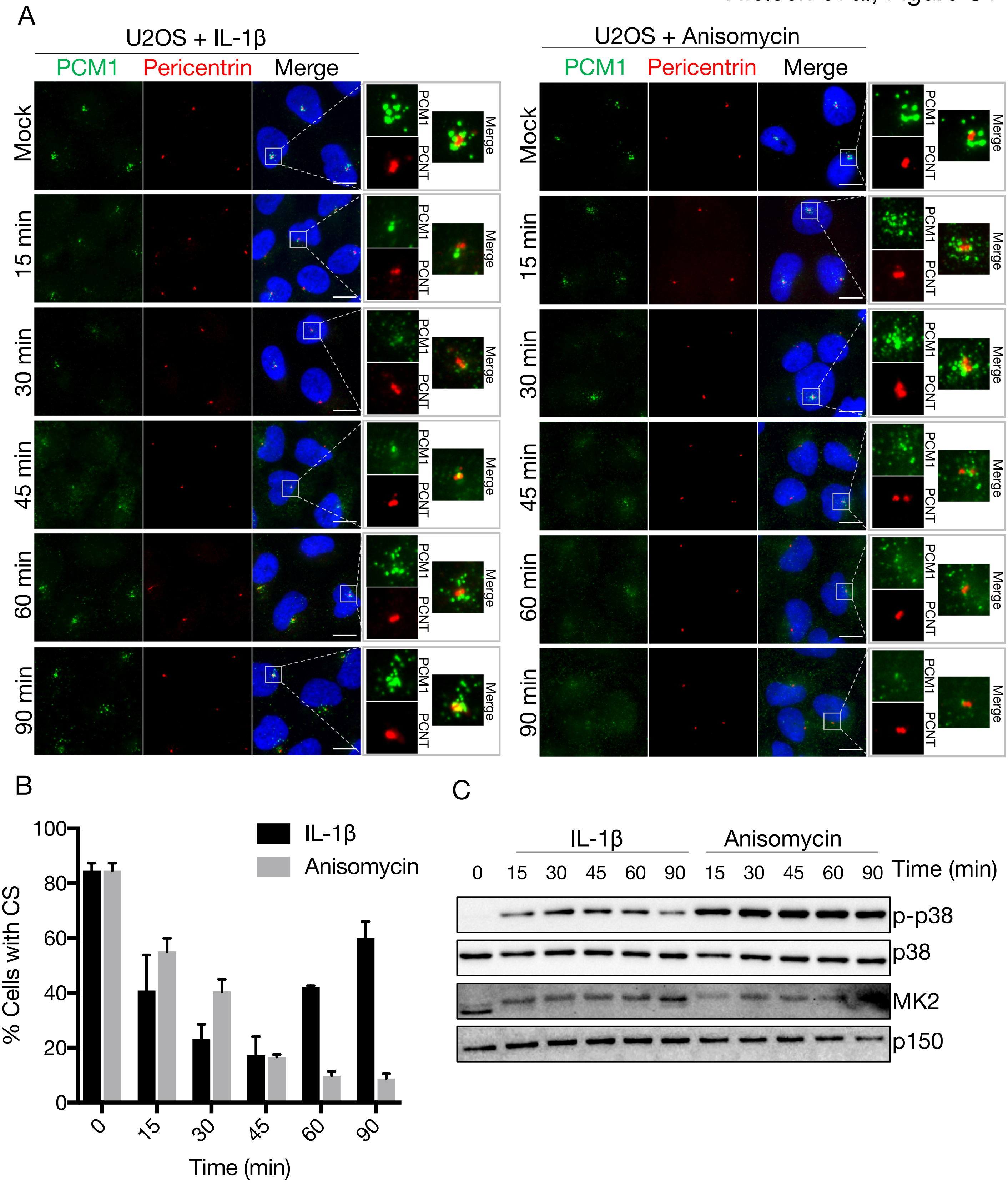

Figure S2.

Sorbitol blocks p38-mediated centriolar satellite depletion in several human cell lines

- **a.** U2OS cells were treated with anisomycin or NaCl (1h) in the indicated combinations. Cells were immunostained with antibodies against PCM1 and pericentrin and counterstained for nuclear content with DAPI. PCNT; Pericentrin. Scale bars, 10 µm.
- **b.** Quantification of (a). At least 100 cells were scored per condition in 3 independent experiments. Bars indicate the mean +/- SEM. P-values were calculated from a one-way ANOVA using Dunnett's correction for multiple testing. ****, P<0.0001, n.s.; not significant. CS; centriolar satellites.
- **c.** U2OS cells were treated with anisomycin or sorbitol (1h) in the indicated combinations. Cells were immunostained with antibodies against CEP131 and counterstained for nuclear content with DAPI.
- **d.** As in (c) except that RPE1 cells were used.
- **e.** As in (c) except that HaCaT cells were used.
- **f.** Lysates of cells from (c), (d) and (e) were analysed by immunoblotting with antibodies against MK2, phospho-HSP27 and p150 (loading control). All scale bars, 10 µm.

Nielsen et al, Figure S2

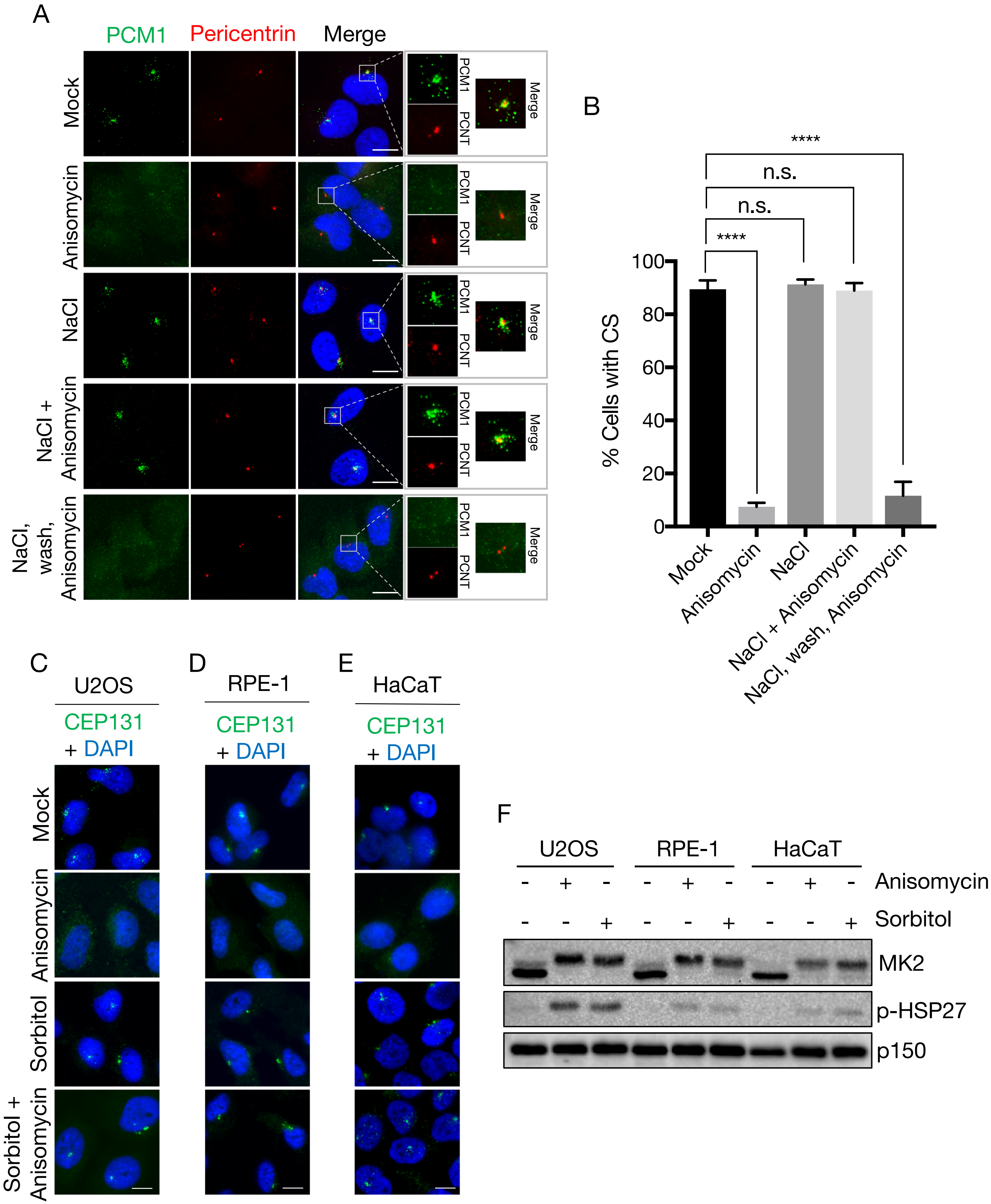

Figure S3.

Effects of sorbitol on centriolar satellite localization

- **a.** U2OS cells were left untreated (mock) or incubated in the presence of sorbitol (1h). Cells were immunostained with antibodies against PCM1 and pericentrin, counterstained for nuclear content with DAPI, and subjected to confocal microscopy.
- **b.** U2OS cells were transfected with control (CTRL) or SSX2IP-targeting siRNAs and treated with anisomycin as indicated.
- **c.** Quantification of (d). At least 100 cells were scored per condition in 3 independent experiments. Bars indicate the mean +/- SEM. P-values were calculated from a one-way ANOVA using Dunnett's correction for multiple testing. n.s.; not significant. CS; centriolar satellites.
- **d.** Lysates from mock treated cells in (b) were analysed by immunoblotting with antibodies against SSX2IP and p150 (loading control).
- **e.** U2OS cells were treated for 3h with nocodazole and for an additional 1h with anisomycin. Cells were immunostained with antibodies against CEP131 and α-tubulin and counterstained for nuclear content with DAPI.
- **f.** U2OS cells were pretreated with sorbitol and taxol for the indicated times and immunostained as in (b). Cells were immunostained with antibodies against CEP131 and γ-tubulin and counterstained for nuclear content with DAPI. PCNT; Pericentrin. All Scale bars, 10 μm.

Nielsen et al, Figure S3

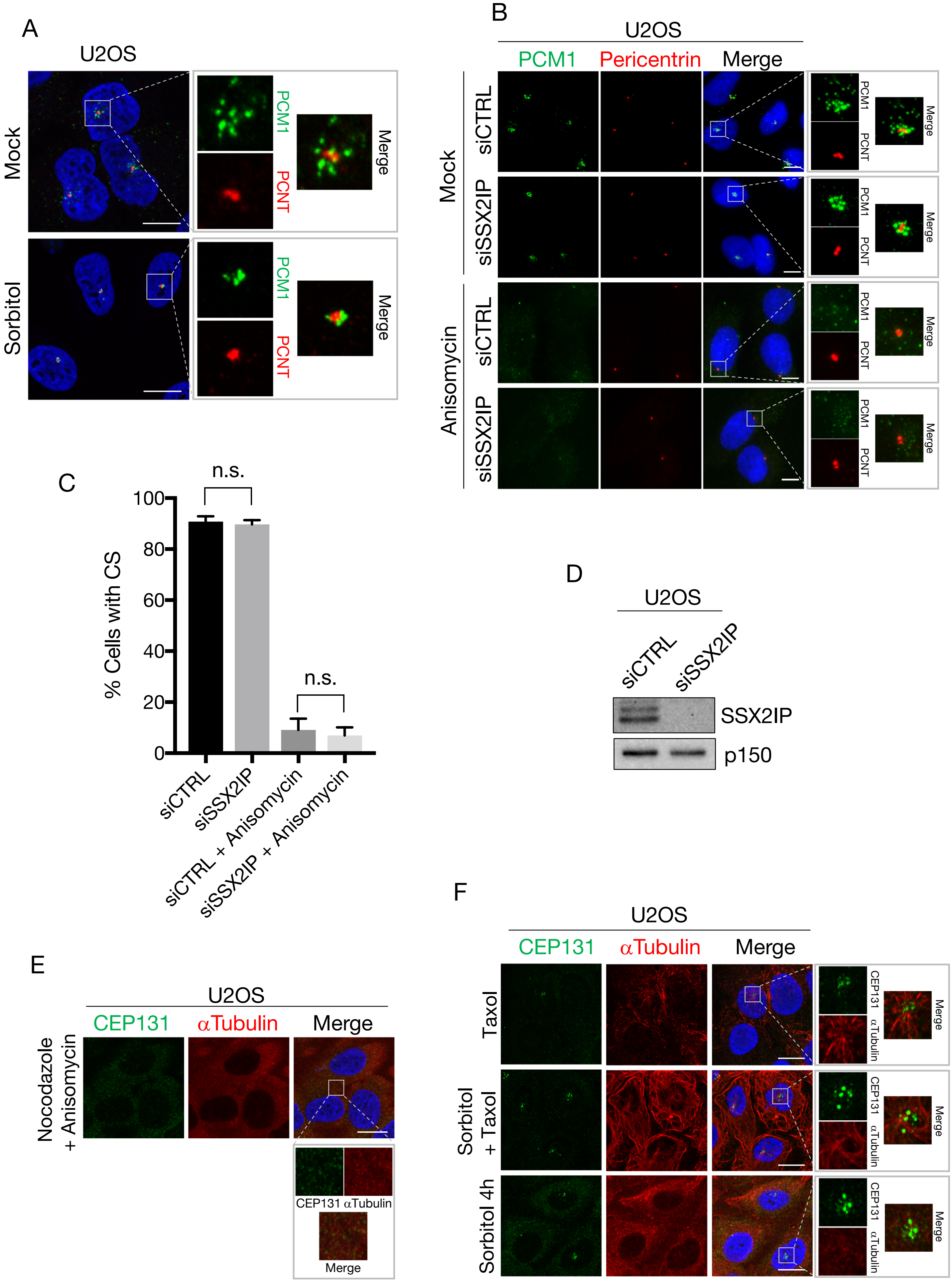

Supplemental movies

Supplementary movie 1

Mobility of centriolar satellites in untreated cells

Time-lapse of U2OS: Flp-In T-Rex GFP-CEP131 cells. Cells were grown in CO_2-independent medium on glass-bottom dishes and imaged for GFP fluorescence over the course of 10 min. Scale bar, 5 μm.

Supplementary movie 2

Mobility of centriolar satellites in sorbitol treated cells

Time-lapse of U2OS: Flp-In T-Rex GFP-CEP131 cells. Cells were grown in CO_2-independent medium on glass-bottom dishes and imaged for GFP fluorescence over the course of 10 min. Scale bar, 5 μm.

Supplementary movie 3

Trajectories of individual centriolar satellites in untreated cells

Time-lapse of U2OS: Flp-In T-Rex GFP-CEP131 cells. Cells were grown in CO_2-independent medium on glass-bottom dishes and imaged for GFP fluorescence over the course of 10 min. Trajectories of individual satellites were assigned using the MOSAIC ImageJ/Fiji plug-in. Scale bar, 5 μm.

Supplementary movie 4

Trajectories of individual centriolar satellites in sorbitol treated cells

Time-lapse of U2OS: Flp-In T-Rex GFP-CEP131 cells. Cells were grown in CO_2-independent medium on glass-bottom dishes and imaged for GFP fluorescence over the course of 10 min. Trajectories of individual satellites were assigned using the MOSAIC ImageJ/Fiji plug-in. Scale bar, 5 μm.
